# RNA interference-mediated depletion of TRPM8 enhances the efficacy of epirubicin chemotherapy in prostate cancer LNCaP and PC3 cells

**DOI:** 10.3892/ol.2022.13211

**Published:** 2022-01-26

**Authors:** Tao Liu, Yixiang Liao, Huangheng Tao, Jinmin Zeng, Gang Wang, Zhonghua Yang, Yongzhi Wang, Yu Xiao, Jiajie Zhou, Xinghuan Wang

Oncol Lett 15: 4129-4136, 2018; DOI: 10.3892/ol.2018.7847

Subsequently to the publication of the above article, the authors have realized that they made some errors during the assembly of Fig. 3A. First, one of the group names, “soCon”, should have been written as “siCON”, which represents the control experiment for TRPM8 without EPI. Secondly, the second flow cytometric plot on the left in the lower row was an inadvertent duplication of the first plot shown in the upper row. However, the authors were able to identify their original data, and the correct version of Fig. 3, showing the correct data for the PC3 / siCon experiment in Fig. 3A, is shown below. Note that the errors in this figure did not affect either the results or the conclusions reported in this study. The authors are grateful to the Editor of *Oncology Letters* for granting them the opportunity to publish this corrigendum, and regret any inconvenience caused to the readership of the Journal.

## Figures and Tables

**Figure 3.**
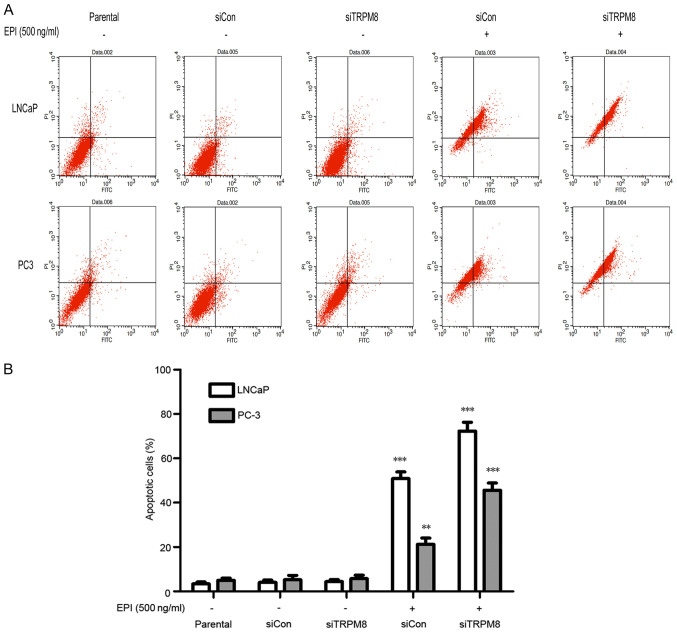
Knockdown of TRPM8 enhanced EPI-induced apoptosis, analyzed by flow cytometry. (A) LNCaP (above) and PC3 (below) cells were incubated with 500 ng/ml EPI for 48 h and then was harvested for apoptosis analysis. (B) The results of the flow cytometry analysis were quantified and expressed in histograms. The figures are representative of three experiments. **P<0.01, ***P<0.001, compared with the parental group. siTRPM8, small interfering RNA targeting transient receptor potential cation channel subfamily M member 8; EPI, epirubicin; CON, negative control.

